# Attenuated *Listeria monocytogenes*: a powerful and versatile vector for the future of tumor immunotherapy

**DOI:** 10.3389/fcimb.2014.00051

**Published:** 2014-05-12

**Authors:** Laurence M. Wood, Yvonne Paterson

**Affiliations:** ^1^Immunotherapeutics and Biotechnology, Texas Tech University Health Sciences CenterAbilene, TX, USA; ^2^Microbiology, Perelman School of Medicine, University of PennsylvaniaPhiladelphia, PA, USA; ^3^University of Pennsylvania School of NursingPhiladelphia, PA, USA

**Keywords:** Listeria monocytogenes, tumor immunotherapy, cancer vaccines, tumor-associated antigens, vaccine vectors and adjuvants

## Abstract

For over a century, inactivated or attenuated bacteria have been employed in the clinic as immunotherapies to treat cancer, starting with the Coley's vaccines in the 19th century and leading to the currently approved bacillus Calmette-Guérin vaccine for bladder cancer. While effective, the inflammation induced by these therapies is transient and not designed to induce long-lasting tumor-specific cytolytic T lymphocyte (CTL) responses that have proven so adept at eradicating tumors. Therefore, in order to maintain the benefits of bacteria-induced acute inflammation but gain long-lasting anti-tumor immunity, many groups have constructed recombinant bacteria expressing tumor-associated antigens (TAAs) for the purpose of activating tumor-specific CTLs. One bacterium has proven particularly adept at inducing powerful anti-tumor immunity, Listeria monocytogenes (Lm). Lm is a gram-positive bacterium that selectively infects antigen-presenting cells wherein it is able to efficiently deliver tumor antigens to both the MHC Class I and II antigen presentation pathways for activation of tumor-targeting CTL-mediated immunity. Lm is a versatile bacterial vector as evidenced by its ability to induce therapeutic immunity against a wide-array of TAAs and specifically infect and kill tumor cells directly. It is for these reasons, among others, that Lm-based immunotherapies have delivered impressive therapeutic efficacy in preclinical models of cancer for two decades and are now showing promise clinically. In this review, we will provide an overview of the history leading up to the development of current Lm-based immunotherapies, the advantages and mechanisms of Lm as a therapeutic vaccine vector, the preclinical experience with Lm-based immunotherapies targeting a number of malignancies, and the recent findings from clinical trials along with concluding remarks on the future of Lm-based tumor immunotherapies.

## Introduction

**Figure 1:**
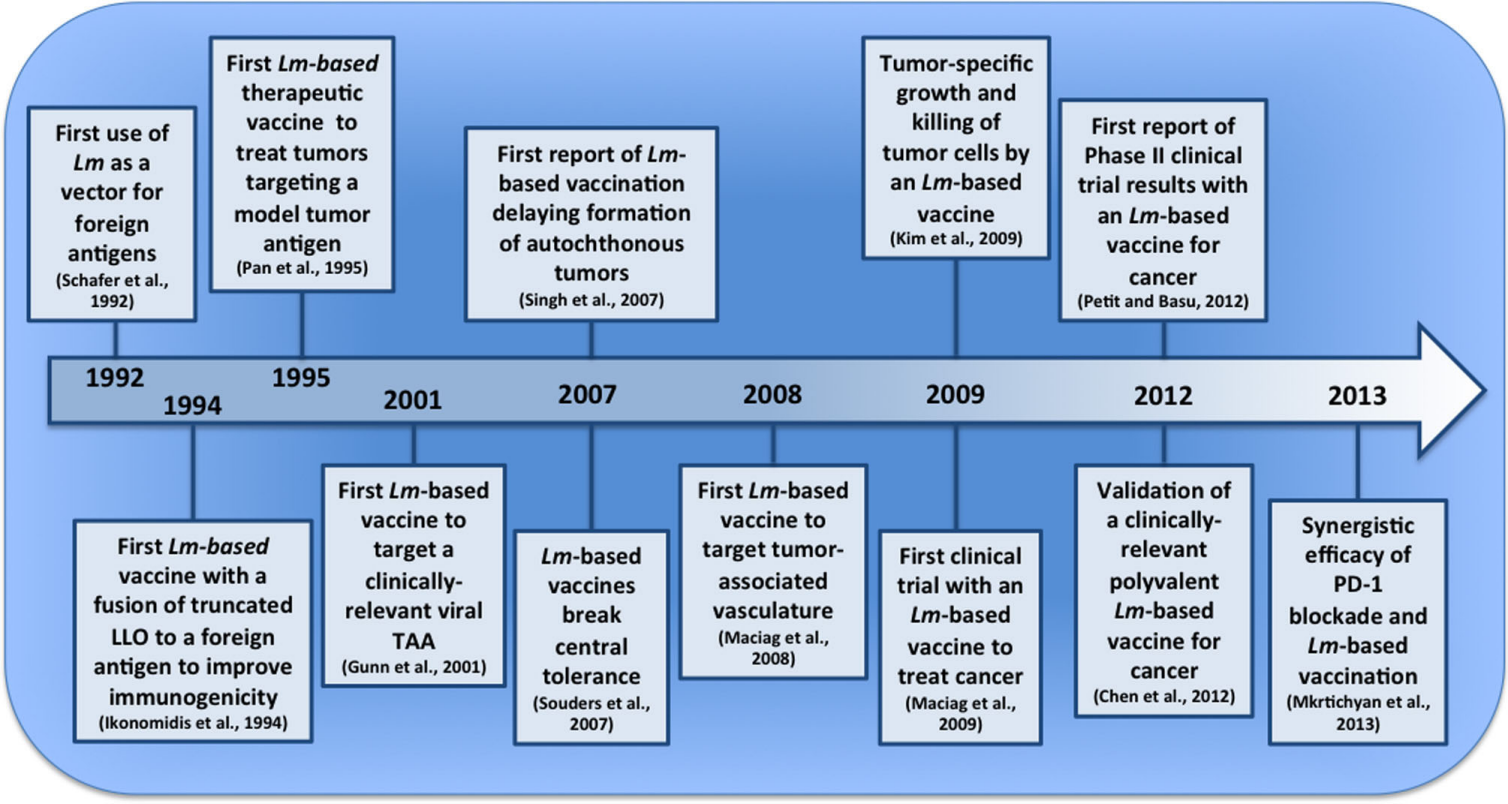
**Pivotal events in the development of *Lm*-based vaccines for tumor immunotherapy**. The last two decades have seen the emergence of *Lm*-based vaccines and their first clinical application for cancer. In this timeline (not to scale), we depict several pivotal events that have led to their current stage of development and point to a future with improved *Lm*-based vaccines and treatment strategies.

Tumor immunotherapy is currently gaining momentum as an invaluable therapeutic strategy for cancers refractory to traditional treatments or those with no effective therapeutic options. The recent FDA-approval of the first antigen-specific tumor immunotherapy, Sipleucel-T for prostate cancer (also known as Provenge), and the introduction of an immune checkpoint modulating therapeutic, Ipilumimab for metastatic melanoma, are part of a new wave of immunotherapies that bring with them the promise of improved efficacy and reduced adverse events in comparison to chemotherapy and radiation (Hodi et al., 2010; Kantoff et al., 2010a). In this review, we will provide a detailed overview of another promising immunotherapeutic approach still in development, *Listeria monocytogenes (Lm)*-based vaccines. This review will cover the important developments leading up to the application of *Lm* in tumor immunotherapy (depicted in Figure 1), the mechanisms governing efficacy by *Lm*-based tumor vaccines, the preclinical and clinical experience with *Lm*-based tumor vaccines, and concluding remarks on their future.

### Attenuated or inactivated bacteria as non-specific tumor immunotherapy

Modern tumor immunotherapy has its beginnings in the late-20th century with the introduction of IL-2 for advanced kidney cancer but the field is influenced by a history of immunization with attenuated or inactivated pathogens that dates back millennia (Rosenberg et al., 1987). From ninth century Asia, where physicians administered an attenuated variola virus to prevent smallpox to the eighteenth century development of the first vaccine for smallpox by Fenner (1993), we have become increasingly successful at employing attenuated and inactivated pathogens that safely and effectively mobilize immunity against infectious diseases. However, the application of a similar approach to mobilize therapeutic immunity against cancer has been complicated by obstacles that still hinder most active tumor immunotherapies. One of the most apparent difficulties of applying a similar strategy is that tumors are self-tissue for which our immune system is tolerant (Hogquist et al., 2005). However, even with this tolerance, anti-tumor immune responses are observed in patients but they are poorly functional and insufficient to eliminate the malignancy (Ahmadzadeh et al., 2009). An early indication for a strategy to overcome this tolerance and enhance anti-tumor immunity came from observations by Vautier in 1813 detailing tumor regression in patients infected with *Clostridium perfingens* (Hall, 1998). In 1851, a Belgian doctor named Didot reported on the successful treatment of terminal cancer patients by deliberately giving them syphilis (Hall, 1998). However, observational evidence and a small study performed by a German scientist Busch suggested that infection with *Streptococcus pyogenes* was the most effective method to induce tumor regressions (Hall, 1998). Building on observations from Vautier and others regarding the clinical benefit of bacterial infection in cancer patients, the first systematic studies were performed by William Coley with heat-inactivated mixtures of bacteria known as Coley's toxins (Coley, 1991). William Coley, a bone surgeon at Memorial Sloan-Kettering in the late nineteenth and early twentieth century, treated his cancer patients with an inactivated bacterial mixture that consisted mainly of *Streptococcus pyogenes* and *Serratia marcescens* in order to activate non-specific anti-tumor immunity. Inoculation with the Coley's toxin or “Coley's vaccine” resulted in a significant number of partial and complete responses in these patients. The advent of radiation therapy brought about the demise of this approach but interest in similar approaches has been gaining acceptance in recent decades (Wiemann and Starnes, 1994; Karbach et al., 2012). In fact, attenuated *Mycobacterium bovis*, specifically the Bacillus Calmette-Guerin (BCG) strain, is currently in use as an immunotherapy for bladder cancer (Herr et al., 1995). Much like Coley's toxins, BCG is utilized as a non-specific immune stimulant.

### Current strategies for activating tumor-specific immunity

Non-specific activation of innate immunity has found some clinical success but the current field is now focused on the development of more effective and specific immunotherapies that activate cytotoxic T lymphocytes (CTL) targeting tumor-associated antigens (TAAs). In fact, the first FDA-approved active tumor immunotherapy, Sipleucel-T, was developed using this CTL-mediated strategy (Kantoff et al., 2010a). To activate TAA-specific CTLs, Sipleucel-T is comprised of a patient's PBMCs that are treated with granulocyte macrophage colony-stimulating factor (GM-CSF) fused to the prostate cancer specific antigen, prostatic acid phosphatase (PAP). The activated, PAP epitope-presenting PBMCs are then administered back to the patient in order to induce CD8^+^ T cells that recognize and lyse prostate tumor cells expressing PAP. The end result of this therapy is increased patient survival of roughly 4 months in those with advanced disease. While the extended lifespan afforded by Sipleucel-T is certainly encouraging, the relatively high cost and labor required to make a treatment specific to each patient makes this option less than ideal for widespread application. Fortunately, there are a number of therapeutic vaccines utilizing attenuated viral and bacterial pathogens as vectors in clinical and preclinical testing that mitigate many of these issues while promising potentially greater efficacy (Paterson et al., 2010; Larocca and Schlom, 2011). These promising attenuated pathogen vectors do come with their own set of advantages and disadvantages as well. Viral vectors such as the commonly used modified vaccinia virus Ankara (MVA) strain have the advantage of delivering genes encoding large antigens that bacterial vectors have difficulty properly secreting (Jacobs et al., 2009; Wood et al., 2011). The major disadvantage of MVA-based and other viral vaccine vectors, however, is that they are no longer able to boost immunity after the second immunization due to induction of neutralizing antibodies (Kundig et al., 1993). To overcome this drawback, recent studies have found some success by boosting with recombinant fowlpox virus vectors that do not develop neutralizing antibodies but induce less robust immune responses (Taylor and Paoletti, 1988; Kantoff et al., 2010b). In contrast, there is either a minor or equivocal impact of preexisting immunity on the efficacy of the two most common bacteria-based vectors for tumor immunotherapy, *Salmonella typhimurium* (*Salmonella*) and *Listeria monocytogenes* (*Lm*) (Tvinnereim et al., 2002; Sevil Domenech et al., 2007, 2008; Leong et al., 2009; Saxena et al., 2009). In addition, the minimal negative impact of preexisting immunity on responses to a delivered antigen by *Lm* can be mitigated with repeated boosting (Leong et al., 2009). The difference between these bacterial vectors becomes readily apparent, however, when comparing their life cycle and subcellular localization. In lymphoid organs, *Salmonella* is taken up into the phagosome of infected macrophages where it remains sequestered and slowly replicates (Haraga et al., 2008). *Lm* is also taken up into phagosomes but is able to escape into the cytosol where it rapidly divides and delivers antigens efficiently to the MHC I presentation pathway (Portnoy et al., 2002). This efficient delivery of antigens to the cytosol by *Lm* results in a more rapid antigen-specific cellular response and greater anti-tumor efficacy in comparison to a *Salmonella* vector expressing the same antigen (Stark et al., 2009). Interestingly, Nishikawa et al. discovered a method to improve *Salmonella* delivery of antigens to the cytosol and induce tumor-specific CTL responses through the use of the Type III secretion system (Nishikawa et al., 2006). While the number of studies is limited, *Lm*-based vaccines for cancer have also demonstrated greater therapeutic benefit in clinical trials as compared to a *Salmonella*-based vaccine (Toso et al., 2002; Maciag et al., 2009; Le et al., 2012; Petit and Basu, 2013). Due to these advantages and others outlined below, *Lm* is one of our most promising vectors available for tumor immunotherapy and the focus of this review.

### Attenuated *Lm* as a powerful vector for targeted tumor immunotherapy

#### Lm infection and host response

*Listeria monocytogenes* is a gram-positive bacterium that is commonly associated with gastrointestinal infections through the consumption of contaminated food. As a gastrointestinal infection, *Lm* infects epithelial cells in the small intestine through the function of a virulence factor known as Internalin A (InlA) (Gaillard et al., 1991). InlA specifically interacts with E-cadherin expressed on the basolateral surface of polarized epithelium to facilitate invasion into a cell (Pentecost et al., 2006). After invading the gut epithelium, *Lm* then migrates to other organs such as the spleen where it is phagocytized by antigen-presenting cells (APCs). Inside the cell, a prfA-mediated virulence gene program in *Lm* facilitates its survival and propagation (Freitag et al., 1993). PrfA is a *Lm*-specific transcriptional activator of many virulence factors such as the pore-forming toxin Listeriolysin O (LLO) and phospholipases that coordinate to compromise the integrity of phagosomal membranes and allow for *Lm* escape into the cytosol of the infected cell (Mengaud et al., 1987). Once it has escaped from the phagosome, *Lm* propels through the cytosol through the action of ActA, a protein that interacts with the Arp2/3 complex to activate the nucleation of actin filaments. It is this cytosolic motility of *Lm* that allows it to impact the cellular membrane with enough force to form a protrusion that is recognized and internalized by a proximal cell, therefore, disseminating the infection (Tilney and Portnoy, 1989).

The host response to infection by *Lm* is evident in both the strong innate response to the intracellular pathogen during an acute infection and the generation of strong adaptive responses to counter future challenges with *Lm*. The innate response to *Lm* is initially mediated by surface toll-like receptors (TLRs) that recognize bacterial pathogen-associated molecular patterns (PAMPs) from *Lm* such as lipoteichoic acid (Machata et al., 2008; Noor et al., 2008). Activation of TLRs by *Lm* results in downstream signaling through the MyD88 adaptor protein and production of proinflammatory cytokines (Edelson and Unanue, 2002). Once phagocytized, *Lm* may then be processed in the phagolysosomal compartment and peptides presented on MHC Class II for activation of *Lm*-specific CD4^+^ T cell responses. Alternatively, *Lm* can escape the phagosome and enter the cytosol where recognition of peptidoglycan by nuclear oligomerization domain-like receptors (NLRs) and *Lm* DNA by the DNA sensor, AIM2, activates inflammatory cascades (Kobayashi et al., 2005; Hasegawa et al., 2006; Park et al., 2007; Sauer et al., 2010; Warren et al., 2010). Once in the cytosol, *Lm* also secretes protein antigens that are processed and presented by MHC Class I molecules to CD8^+^ T cells for robust induction of *Lm*-specific CTL responses that provide protection against a subsequent exposure (Brunt et al., 1990). This combination of inflammatory responses and efficient delivery of antigens to the MHC I and MHC II pathways makes *Lm* a powerful vaccine vector.

#### Lm as a vaccine vector for delivery of foreign antigens

The ability of Lm to induce strong cellular immune responses has led to its use for decades as a tool to dissect the mechanisms of immune memory formation and function (North, 1973; Stolpmann et al., 1985; Shedlock et al., 2003; Shen et al., 2003). However, it was not until several studies in the early 1990s that the enormous translational potential of Lm as an inducer of antigen-specific CTLs was realized (Schafer et al., 1992; Ikonomidis et al., 1994; Frankel et al., 1995; Pan et al., 1995a,b). In the initial study, Paterson and colleagues constructed recombinant strains of Lm that expressed a foreign antigen, *E. coli* β-galactosidase (β-gal) (Schafer et al., 1992). When mice were immunized either parenterally or orally with these constructs, β-gal-specific CTLs were induced and delayed type-hypersensitivity reactions to β-gal were observed in mice receiving the recombinant Lm strain but not a wild-type Lm strain. Building on this success, Paterson and colleagues further developed and refined the Lm vaccine platform with a construct that expressed a viral antigen, influenza nucleoprotein (NP) (Ikonomidis et al., 1994). However, this Lm vaccine sought to improve the processing and presentation of the foreign antigen by use of a strategy still used in Lm-based vaccines to this day. Unlike the β-gal Lm construct, the Lm-NP construct expressed the antigen as a fusion protein with the first 420 amino acids of Listeriolysin O. This LLO-NP fusion protein lacked the cholesterol-binding domain at the C-terminus of LLO necessary for pore formation and was, therefore, detoxified while still containing the signal sequence at the N-terminus allowing for efficient secretion (Michel et al., 1990). The rationale behind this approach was that the improved secretion of the foreign antigen into the cytosol of infected cells would increase the efficiency of processing and presentation to match the immunogenicity of endogenous Lm antigens. In fact, Lm-NP infected cells were able to efficiently process and present NP peptides for recognition by NP-specific CTLs. Consequently, Lm-NP constructs induced a highly functional CTL response against NP that was dependent on the ability of the constructs to deliver antigen to the cytosol, as LLO-deficient constructs were ineffective. The major advancement from this work of improving the processing and presentation of foreign antigens by Lm-based vaccines allowed for future application of this technology in CTL-mediated tumor immunotherapy.

#### Initial application of Lm vaccines in tumor immunotherapy

After confirming the potential of *Lm*-based vectors to induce robust CTL responses against foreign antigens, Paterson and colleagues applied this technology to the then nascent field of tumor immunotherapy (Pan et al., 1995a). Much like infected cells, tumor cells can be recognized and lysed by antigen-specific CTLs (Freedman et al., 1972). Therefore, to assess the potential for *Lm*-based vaccines to induce effective anti-tumor CTL responses, the team utilized the previously constructed *Lm*-NP vaccine strain along with tumor cell lines that expressed NP as a model tumor antigen (Pan et al., 1995a). When mice were vaccinated with the *Lm*-NP vaccine, it provided prophylactic protection against tumor formation after challenge. In addition, after first allowing for tumors to form and establish, therapeutic intervention was effective at reducing tumor volume. This regression by *Lm*-NP correlated with infiltration of T cells into the tumor after vaccination and was abrogated after antibody-mediated depletion of T cells. A subsequent study by Jensen and colleagues also found *Lm*-based vaccines to be effective as a prophylactic against cottontail rabbit papilloma virus (CRPV) induced papillomas, a model for virus-induced malignant transformation (Brandsma, 1994). After previous success generating virus-specific CTL responses with recombinant *Lm*, they developed a novel *Lm* strain expressing the CRPV E1 antigen, E1-r*Lm*, that induced cellular immune responses against E1 and protected the rabbits from papilloma formation (Shen et al., 1995; Slifka et al., 1996; Jensen et al., 1997). Therefore, the summation of these studies validated *Lm* as a viable platform for the delivery of TAAs in both a prophylactic and therapeutic setting. The promise demonstrated by these initial vaccines resulted in the development of numerous *Lm*-based immunotherapies, many outlined in this review, that continues to this day.

## Mechanisms of *Lm*-based vaccine vectors for tumor immunotherapy

**Figure 2:**
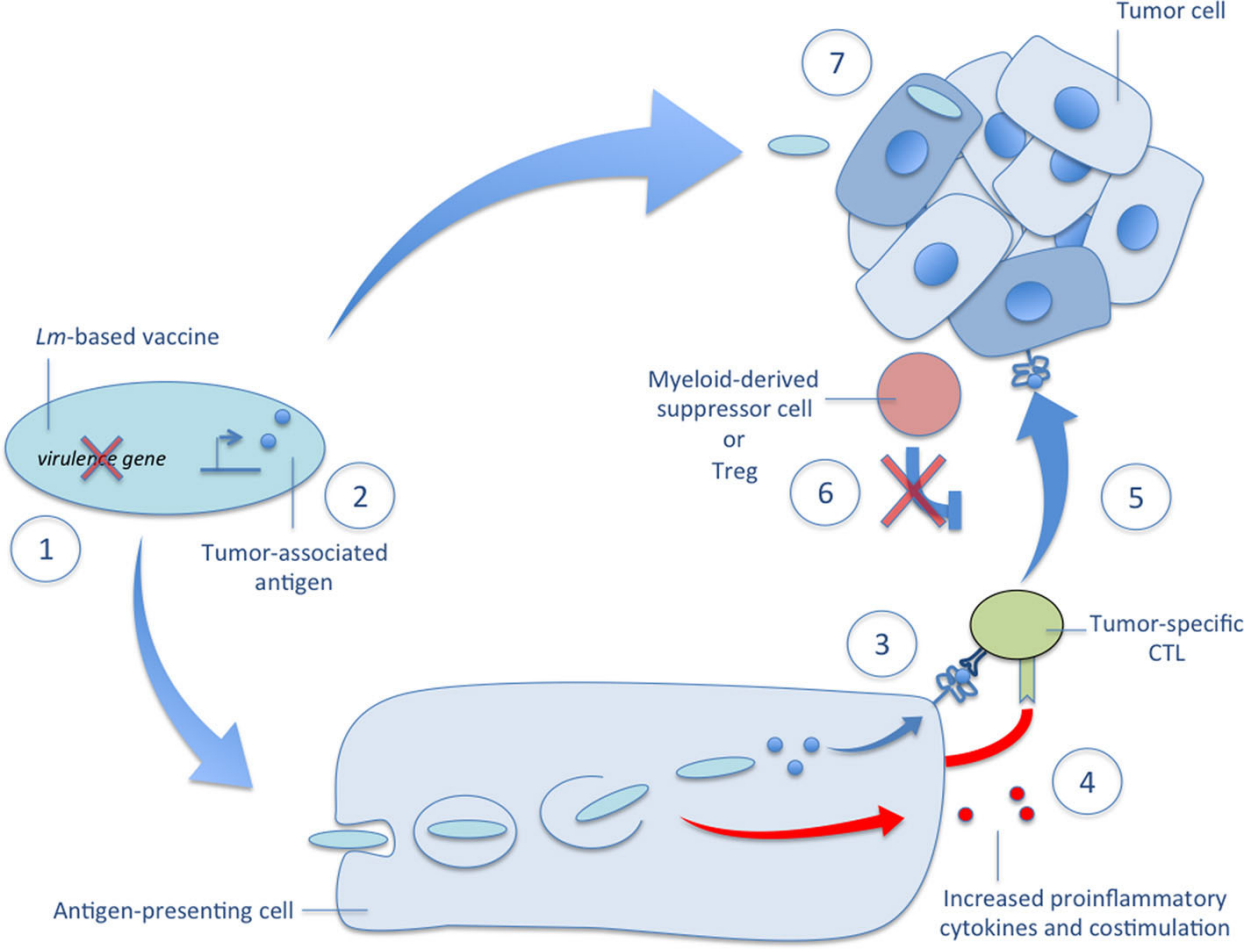
**Mechanisms of *Lm*-based vectors in tumor immunotherapy**. Several mechanisms governing the efficacy of *Lm*-based immunotherapies are depicted in the accompanying figure. (1) There are several very effective methods of attenuation available to construct a highly immunogenic *Lm*-based vector usually involving deletion of one or more virulence genes. (2) Once attenuated, *Lm*-based vectors are highly versatile producers of tumor-associated antigens and other therapeutic proteins. (3) After administration, *Lm*-based vaccines selectively infect antigen-presenting cells, escape the phagosome, and secrete tumor-associated antigens that are delivered to a high-efficiency processing and presentation pathway for activation of tumor-specific CTLs. (4) The detection of *Lm*-derived PAMPs facilitates tumor-specific CTL activation through the upregulation of costimulatory molecules and the secretion of proinflammatory cytokines. (5) The resultant effect of *Lm*-based vaccination is the ability to break central tolerance and produce therapeutic anti-tumor CTL responses against self TAAs. (6) This tumor-specific CTL response is further aided by reduced tumor-associated immunosuppression as evidenced by diminished functionality and lower numbers of Tregs and MDSCs in the tumor microenvironment after *Lm*-based vaccination. (7) In addition to activation of tumor-specific CTL responses, recent reports demonstrate the ability of *Lm*-based vaccines to target primary and metastatic tumors for infection and directly kill tumor cells. Each of these mechanisms of *Lm*-based vaccines highlight their powerful potential as vectors for tumor immunotherapy and their versatility in terms of construction and efficacy.

Early studies demonstrated the powerful potential of *Lm*-based immunotherapies for cancer but the mechanisms governing their efficacy are still under investigation. We describe, in detail below and depicted in Figure 2, important advances in our understanding of *Lm*-based immunotherapy efficacy that continue to guide its development.

### Enhanced processing and presentation of *Lm*-delivered antigens

The initial rationale for the use of *Lm* as a vaccine vector was to harness its ability to deliver antigens efficiently to both the MHC Class I and II presentation machinery and induce robust T-cell responses to Lm-derived antigens (Naher et al., 1985; Lukacs and Kurlander, 1989; Brunt et al., 1990; Schafer et al., 1992; Hsieh et al., 1993). However, recent studies suggest that the robust CD8^+^ T cell responses to *Lm*-derived antigens are not simply due to proficient cytosolic delivery. In fact, *Lm*-derived antigens appear to be processed and presented on MHC Class I by a dedicated, high-efficiency pathway (Wolf and Princiotta, 2013). In a study by Wolf and Princiotta, *Lm*-based vectors were constructed that expressed influenza NP fused to the first 100 amino acids of ActA in order to allow for proper secretion of the antigen (Wolf and Princiotta, 2013). However, one construct expressed the fusion protein with an arginine at the N-terminus of NP to give the protein a half-life of 10 min while the unmodified fusion protein had a half-life of several days. Interestingly, APCs infected with either *Lm*-based construct presented an epitope peptide from the antigen on MHC Class I with the same efficiency. This result suggests that *Lm*-derived antigens are presented with high-efficiency independent of the protein half-life. This high-efficiency presentation pathway was exclusive for *Lm* as a viral vector delivering the same antigens resulted in presentation that directly correlated with protein half-life. Therefore, this evidence provides additional rationale for the use of *Lm* as a CTL vaccine vector and elucidates an additional mechanism for the robust T cell responses induced by *Lm*-based immunotherapies.

### Activation of innate immunity by *Lm*-derived PAMPs

#### Detoxified listeriolysin O (dtLLO)

Listeriolysin O is a cholesterol-dependent cytolysin that, upon secretion by Lm, binds to cholesterol-containing membranes and forms a barrel resulting in a membrane pore (Shatursky et al., 1999). LLO-mediated pore formation results in reduced phagosome membrane integrity and aids the escape of Lm from the phagosomes of APCs (Geoffroy et al., 1987; Tilney and Portnoy, 1989; Beauregard et al., 1997). While the acidic environment of the phagosome increases the lytic activity of LLO for this purpose (Glomski et al., 2002), others have also found extracellular secretion of LLO by Lm that directly lyses T cells (Carrero et al., 2004). In a therapeutic setting, LLO has proven a powerful adjuvant but only after this lytic activity is reduced though “detoxification” with mutation or truncation of its cholesterol-binding domain (Michel et al., 1990). The first use of a detoxified LLO (dtLLO) in a tumor immunotherapy setting was in the context of a genetic fusion with a tumor-specific antigen (Pan et al., 1995a). Fusion of the antigen to dtLLO and secretion by Lm was found to significantly enhance anti-tumor immune responses in comparison to an Lm vector that secreted only the tumor antigen (Gunn et al., 2001). The likely mechanisms for this adjuvancy effect of dtLLO are several. The N-terminus of dtLLO contains an N-degron, a destabilizing residue that mediates processing by the Ub-proteasome machinery (Schnupf et al., 2006, 2007). Therefore, it was hypothesized that tumor antigens genetically fused to the C-terminus of dtLLO may have enhanced Ub-proteasome-mediated processing and presentation by APCs for activation of antigen-specific CTLs. More recently, several studies have inspected the potential for dtLLO to function as a PAMP and stimulate the necessary proinflammatory responses for effective anti-tumor adaptive immune responses (Peng et al., 2004, 2007; Wallecha et al., 2013b). A study by Wallecha et al found that purified dtLLO administered as a fusion protein with human papilloma virus type 16 E7 protein (HPV16 E7), a TAA expressed by HPV-transformed malignancies, or unfused in a mixture with HPV16 E7 can augment anti-E7 CD8^+^ T cell responses and therapeutically impact growth of murine tumors that express E7 (Wallecha et al., 2013b). To determine if the mechanism of adjuvancy was through PAMP-like activity, bone marrow-derived dendritic cells (BMDC) from wild-type mice were stimulated *in vitro* with dtLLO. dtLLO stimulation resulted in induction of proinflammatory cytokines such as IL-12 and increased expression of markers associated with BMDC maturation. While previous studies have suggested that PAMP-like activity of LLO and other cytolysins is dependent on TLR4 (Park et al., 2004), dtLLO treatment resulted in TLR4-independent induction of transcript for proinflammatory cytokines and costimulatory molecules suggesting additional mechanisms for this activity. These results suggest that the fusion or conjugation of dtLLO to antigen is likely not required to improve immunogenicity as previous studies with DNA vaccines suggested but this is yet to be determined in an Lm-based vaccine approach (Peng et al., 2007).

#### ActA

ActA production by Lm is essential to its cytoplasmic motility and cell-to-cell spread due to its ability to nucleate actin through the Arp2/3 complex (Welch et al., 1998). However, much like dtLLO, it is a powerful adjuvant as its fusion to *Lm*-secreted antigens results in enhanced immunogenicity (Sewell et al., 2004a, 2008; Souders et al., 2007). In work published by Sewell et al, secretion of HPV16 E7 fused to ActA resulted in significantly enhanced immunogenicity of E7 resulting in effective anti-tumor immune responses (Sewell et al., 2004a). The use of ActA as an adjuvant is not only restricted to *Lm*-based vaccines, as it also enhances antigen-specific anti-tumor immune responses in DNA and protein vaccine formulations (Wood et al., 2010). The promising potential of ActA in *Lm*-based vaccines was demonstrated by its ability to activate highly effective anti-tumor immune responses even to tolerized antigens (Souders et al., 2007; Sewell et al., 2008). Fusion of ActA to an antigen may increase immunogenicity through increased antigen processing and presentation due to the presence of several putative PEST domains in the N-terminus of ActA. However, ActA is a relatively stable protein (Moors et al., 1999). In support of an alternative mechanism, a subsequent study suggests the adjuvant properties of ActA are independent of fusion to the antigen (Sewell et al., 2004b; Wood et al., 2010). In fact, ActA protein appears to have PAMP-like properties due to its ability to induce proinflammatory cytokines and maturation of BMDCs (Wood et al., 2010).

#### Cyclic di-Amp

The discovery of cyclic di-AMP as the Type I interferon (IFN)-inducing molecule by *Lm* was critical to our understanding of the host response to *Lm* (Woodward et al., 2010; Schwartz et al., 2012; Witte et al., 2013). Cyclic di-AMP is a small dinucleotide secreted by *Lm* once it escapes the phagosome of an infected cell and enters the cytosol. Within the cytosol, *Lm*-derived cyclic di-AMP activates a mitochondrial transmembrane receptor, STING, leading to Type I IFN production (Ishikawa and Barber, 2008). Type I IFN induced by *Lm* and other intracellular facultative bacteria can increase their acute pathogenicity and recent evidence suggests it may even hinder formation of adaptive immunity to *Lm* (Auerbuch et al., 2004; O'connell et al., 2004; Archer et al., 2014). However, there is a growing body of evidence emphasizing the importance of Type I IFN signaling in tumor immunosurveillance and the formation of effective anti-tumor adaptive immunity (Diamond et al., 2011; Fuertes et al., 2011). Therefore, methods that modulate cyclic di-AMP in *Lm*-based tumor immunotherapy may be promising but will require a delicate balance between its ability to impair or enhance anti-tumor immunity.

### Overcoming central tolerance to TAAs

The preclinical success of *Lm*-NP in mouse tumor models presaged the current promising results of *Lm*-based vaccines targeting tumor-associated viral antigens in clinical trials (Maciag et al., 2009; Petit and Basu, 2013). It is likely that some of this success is due to the targeting of a viral antigen for which there is no central tolerance to limit CTL responses. However, effective targeting of self-TAAs is currently a necessity in tumor immunotherapy as the number of virus-associated malignancies containing viral TAAs is unknown. Therefore, in order to determine if *Lm*-based immunotherapies can effectively overcome central tolerance to self-TAAs, an E6/E7 transgenic mouse was developed with expression of HPV16 E6 and E7 oncogenes under the control of the thyroglobulin promoter to drive expression in the thyroid and also in medullary thymic epithelial cells (Souders et al., 2007). HPV16 E6 and E7 expression in the thyroid results in hyperplasia starting at 2 months of age with malignant transformation evident by the formation of papillary carcinomas starting at 6 months of age. Initial work in this E7-tolerized mouse model found that *Lm*-LLO-E7 vaccination was able to break central tolerance and induce regression of implanted TC.1 tumors. TC.1 tumors stably express HPV16 E6 and E7 along with H-ras and are a common model for preclinical immunotherapies targeting HPV-induced cancer (Lin et al., 1996). Further studies determined that *Lm*-LLO-E7, along with an additional *Lm*-based immunotherapy secreting E7 fused to ActA, *Lm*-ActA-E7, were also effective at reducing the development of autochthonous thyroid tumors (Sewell et al., 2008). However, elimination of TC.1 tumors by *Lm*-LLO-E7 was less effective than in wild-type mice. The reason for this reduced efficacy appeared to not be due to increased numbers of T regulatory cells (Tregs) but the generation of lower avidity CTLs in the E6/E7 transgenic mouse as compared to the wild-type mouse after *Lm*-LLO-E7 vaccination. Nevertheless, the demonstration of anti-tumor efficacy in a tolerized animal model provided the rationale for future *Lm*-based immunotherapies to target self-TAAs such as Her2/neu and VEGFR-2 (Singh et al., 2005; Singh and Paterson, 2006a,b, 2007a,b; Seavey et al., 2009b).

### Reduction of tumor-associated immunosuppression

Immunosuppression in the tumor microenvironment is a daunting obstacle that limits the success of current tumor immunotherapy (Singh and Paterson, 2007b; Duraiswamy et al., 2013). It is mediated by a number of suppressive cell types such as Tregs and signaling molecules that limit immune effector cell function (Hussain and Paterson, 2004; Blank et al., 2005). Interestingly, *Lm*-based vaccines alone can mitigate tumor-associated immunosuppression (Wallecha et al., 2013a). Hussein and Paterson demonstrated that Tregs are significantly decreased upon administration of *Lm*-based vaccines that secrete TAAs fused to truncated LLO (Hussain and Paterson, 2004). Tregs in the tumor microenvironment produce anti-inflammatory cytokines such as IL-10 and TGF-β that impair T cell responses and directly inhibit the function of APCs through interaction with the inhibitory receptor, CTLA-4 (Wing et al., 2008). Unexpectedly, an isogenic *Lm*-based vaccine that expressed and secreted only the TAA actually increased the number of Tregs within the tumor microenvironment and demonstrated reduced anti-tumor efficacy (Gunn et al., 2001). Therefore, it is possible that the PAMP-like activity of dtLLO is a major contributor to the reduction of tumor-associated immunosuppression by *Lm*-based vaccines. Subsequent studies have additionally confirmed this reduction in the number of tumor-associated Tregs after *Lm*-based vaccination (Guirnalda et al., 2013). In addition to Tregs, myeloid-derived suppressor cells (MDSCs) can also mediate immunosuppression in tumor-bearing patients (Almand et al., 2001). MDSCs produce anti-inflammatory cytokines and work in concert with Tregs to inhibit CTL responses (Yang et al., 2006). Production and secretion of peroxinitrite by MDSCs results in direct modification of the TCRs of tumor-infiltrating CTLs and limit their ability to recognize MHC Class I presented TAA peptides (Nagaraj et al., 2007). In recent work by Wallecha and colleagues, *Lm*-based vaccines expressing dtLLO-fused antigens reduced not just the number of tumor-infiltrating MDSCs and Tregs but also reduced their suppressive activity (Wallecha et al., 2013a). This finding of reduced tumor-infiltrating MDSCs and Tregs after *Lm*-based immunotherapy was also confirmed in a recent study by Mkrtichyan et al. (2013).

In addition to suppressive cell types in tumors, the function of tumor-specific infiltrating CTLs can also be hampered by expression and ligation of inhibitory receptors on their surface. One common inhibitory receptor expressed on tumor-infiltrating CTLs is programmed death 1 (PD-1), which is activated by its ligands PD-L1 and PD-L2 expressed by tumor cells (Liu et al., 2003; Blank et al., 2005). Activation of PD-1 on T cells results in their gradual loss of function as demonstrated by reduced proinflammatory cytokine production and lytic activity (Wherry, 2011). Interestingly, in a recent study by Yoshimura et al, *Lm*-vaccine treatment resulted in reduced expression of PD-1 by CD8^+^ T cells in the metastases-bearing livers of mice (Olino et al., 2012). A similar downregulation was not observed on CD8^+^ T cells in secondary lymphoid organs of the same mice, however, suggesting that this effect was metastases and/or tumor-specific.

### Highly attenuated but immunogenic *Lm* vaccine strains

In order to develop *Lm* vaccines for the clinic, the use of virulent strains of the pathogen would be unacceptable for patients that are already likely in an immunosuppressed state (Golub et al., 1974). Fortunately, there are established methods of *Lm* attenuation that may not only increase their safety profile but actually enhance immunogenicity of antigens expressed by *Lm.*

#### Deletion of Lm virulence genes

In order to accomplish stable attenuations of *Lm* vaccine strains, groups have employed strains containing deletions of genes involved in *Lm* virulence. The most common *Lm* vaccine strain, XFL-7, lacks the *Lm* transcriptional activator PrfA (Gunn et al., 2001). PrfA regulates the expression of numerous virulence factors such as LLO, therefore, strains lacking the *prfA* gene are highly attenuated due to their inability to escape into the cytosol and avoid phagolysosomal destruction (Freitag et al., 1993). However, in order to generate robust CTL responses, an effective *Lm* vaccine strain must gain access to the cytosol to deliver antigens. Therefore, XFL-7-based *Lm* vaccines are transformed with episomal plasmids encoding the *prfA* gene along with an antigen (Gunn et al., 2001). The introduction of this episomal plasmid complements the deficiency in *prfA*, however, the expression is not sufficient for XFL-7 to regain full virulence and is, therefore, attenuated. Subsequent studies have focused on the development of *Lm* strains with more targeted deletion of virulence factors that are not required for the development of robust immune responses. One such virulence gene, *actA*, is not necessary for the initial infection of *Lm* into a host cell or entry into the cytosol but is required for cell-to-cell spread of the bacterium (Brockstedt et al., 2004). While ActA-specific immunity is observable after an *Lm* infection, these responses are dispensable to the clearance of the bacterium, likely due to the membrane-bound nature of ActA hampering MHC Class I accessibility (Darji et al., 1998). Deletion of *actA* results in a highly attenuated *Lm* strain with an LD50 of 2 × 10^8^ CFUs as compared to 10^5^ CFUs by wild-type *Lm* while still retaining immunogenicity (Brundage et al., 1993; Starks et al., 2004). In fact, *actA*-deficient *Lm* expressing the model tumor antigen, chicken ovalbumin (OVA), is able to induce a more effective anti-tumor response than a wild-type strain of *Lm* expressing the same antigen (Starks et al., 2004). Therefore, attenuation of *Lm* may not just serve the purpose of improving its safety profile but it is also a rational strategy to increase immunogenicity to *Lm*-expressed TAAs. Further advancement in the development of attenuated *Lm* vaccine strains by Brockstedt and colleagues employed deletion of an additional virulence gene, internalin B (*inlB*) in an *actA*-deficient *Lm* strain (Brockstedt et al., 2004). The rationale for the deletion of *inlB* in this background was to construct a vaccine vector that exclusively infects antigen-presenting cells as InlB mediates infection of non-phagocytic cells (Gaillard et al., 1991). This was sought as a strategy to minimize toxicity from the infection, especially to the liver where toxicity is observed due to *Lm* infection of hepatocytes, while maximizing uptake of the vector to professional phagocytic antigen-presenting cells. Deletion of *inlB* in an *actA* deficient strain led to reduced capacity to infect both mouse and primary human hepatocytes and enhanced clearance from the liver of infected mice in comparison to a wild-type strain and the *actA* only deficient strain. Furthermore, deletion of *inlB* in the *actA* deletion strain that expressed the murine colon TAA, gp70, resulted in increased immunogenicity in comparison to *actA* deficiency alone as observed by significantly greater gp70-specific CTL responses, reduced metastatic burden with CT26 colon carcinoma tumors, and increased survival (Olino et al., 2012). The impressive immunogenicity and efficacy of the Δ*plcB* Δ*actA Lm* vaccine strain preclinically has led to its introduction into clinical trials for multiple cancers (Le et al., 2012). However, the deletion of a virulence gene such as *inlB* to limit infection of non-phagocytic cells may be eliminating additional mechanisms of anti-tumor efficacy by *Lm*-based vaccines (Kim et al., 2009). In addition to *inlB*, other studies have focused on deletion of *plcB*, another virulence gene of *Lm* encoding phospholipase C (PC-PLC), in the background of an *actA* deficient *Lm* vaccine strain (Angelakopoulos et al., 2002). PC-PLC is the broad-range phospholipase C of *Lm* that plays an important role in the breakdown of double-membrane vacuoles encountered during cell-to-cell spread by *Lm* (Vazquez-Boland et al., 1992; Smith et al., 1995). Deletion of *plcB* results in a highly attenuated *Lm* with reduced ability to spread cell-to-cell but also reduced toxicity as intracerebral infection is greatly reduced (Schluter et al., 1998). Deletion of both *plcB* and *actA* still allows for the induction of robust T cell responses in mouse models (Darji et al., 2003; Lenz et al., 2008) and a Δ*plcB* Δ*actA* strain has demonstrated safety in a human clinical trial (Angelakopoulos et al., 2002).

#### Killed but metabolically active (KBMA) Lm

One of the earliest methods for safely inducing prophylactic immunity to pathogens was through the use of inactivated or killed forms of the same pathogen, commonly through heating (Smith and Little, 1926). While this method of inactivation is sufficient for induction of humoral responses to pathogen-associated immunogens, it is an ineffective approach to induce CTL-mediated immunity against *Lm*-derived antigens (Schafer et al., 1992). This lack of CTL-mediated immunity with heat-killed *Lm* (HKLM) is likely due in part to an inability to escape from the phagosome into the cytosol to deliver antigens to the MHC Class I pathway (Brunt et al., 1990). Additionally, a study by Bahjat et al. demonstrates that HKLM infection can also actively suppress cell-mediated immunity through induction of IL-10 in a MyD88-dependent manner (Bahjat et al., 2009). Therefore, a new method of inactivation was developed to provide the safety advantages of a killed vaccine but also maintain efficient delivery of antigens to the MHC Class I presentation machinery and induce the necessary inflammatory responses to provide immunogenicity. This new type of inactivation produced an *Lm* that was killed but metabolically active (KBMA) (Brockstedt et al., 2005; Skoberne et al., 2008). In order to achieve this method of inactivation, a recombinant vaccine strain of *Lm* that contains deletions of the *uvrAB* genes involved in nucleotide excision repair is treated with the DNA-intercalating agent psoralen and then DNA-psoralen crosslinking is induced with exposure to UV irradiation. The result is a bacterium that is unable to replicate and is functionally killed due to DNA crosslinking but still able to transcribe encoded genes and produce and secrete protein antigen. Importantly, KBMA *Lm* is also capable of escaping from phagosomes and delivering secreted antigens to the cytosol for MHC Class I processing and presentation (Dubensky et al., 2012). Vaccination with a KBMA *Lm* targeting a model TAA has also been shown provide comparable anti-tumor efficacy to a live-attenuated *Lm* strain but with slightly reduced antigen-specific CD8^+^ T cell responses (Skoberne et al., 2008).

### Methods for the expression of foreign antigens by *Lm*

Expression of antigens by *Lm* can be of episomal origin, but expression can also be from the *Lm* chromosome. Plasmid-based strategies have the advantage of multicopy expression, which may be more efficacious in terms of the amount of antigen protein expressed, but rely on complementation for the maintenance of the plasmid *in vivo*. The retention of plasmid by *Lm in vivo* in one engineered *Listeria* strain is achieved by the complementation of the *prfA gene* from the plasmid in a *prfA* negative mutant *Lm* background (Gunn et al., 2001). Without *prfA* complementation, this mutant *Lm* cannot escape the phagosome and is destroyed by macrophages and neutrophils. As a result, it cannot grow intracellularly or present antigens to the immune system. In early studies it was found that including a copy of *prfA* in the plasmid ensures the *in vivo* retention of the plasmid in *Lm* (Pan et al., 1995a,b). Another approach is based on the *in vitro* and *in vivo* complementation of D-alanine racemase in both *E. coli* and *Lm* strains deficient in D-alanine racemase (*dal*) and D-alanine amino transferase (*dat*) (Verch et al., 2004; Wallecha et al., 2009; Shahabi et al., 2011). This *Lm* vaccine strain, *Lmdd*, has the advantage that it is devoid of antibiotic selection markers. In order to further improve the safety profile of *Lmdd*, current constructs contain a deletion of the virulence gene *actA* resulting in the *LmddA* strain (Wallecha et al., 2009; Ishizaki et al., 2010).

Chromosomal integration techniques can utilize either a phage-based system, with a site-specific integrase to integrate a gene into the genome (Camilli et al., 1993; Lauer et al., 2002) or allelic exchange into a known chromosomal locus (Gunn et al., 2001; Mata et al., 2001). Recombinant strains based on chromosomal integration have been shown to be somewhat more virulent (Gunn et al., 2001) than similar episomal recombinants and are thus less suitable to be used as human vaccines backbones without further attenuation. This has been achieved by the deletion of virulence genes such as *actA* and *inlB* from the *Lm* chromosome, which in combination limit *Lm* growth in the liver, a principal target organ of infection by the wild type organism (Brockstedt et al., 2004).

### *Lm* tropism for primary and metastatic tumors

One of the most promising aspects of *Lm* as a live vaccine vector is its ability to specifically target and thrive within primary and metastatic tumor lesions. Initially documented by Yu et al., recent work has confirmed the tumor-homing properties of *Lm* (Yu et al., 2004; Kim et al., 2009; Quispe-Tintaya et al., 2013). In a study by Gravekamp and colleagues, an *Lm*-based vaccine delivering a portion of the melanoma-associated antigen (MAGE)-b TAA, *Lm*-LLO-Mage-b311-660, along with an empty vector control, *Lm*-LLO, were found to infect both the murine 4T1 mammary carcinoma cell line and the human MCF7 breast tumor cell line *in vitro* (Kim et al., 2009). *In vivo*, *Lm*-LLO-Mage-b311-600 accumulated in primary 4T1 breast tumors as well as 4T1 metastatic lesions in the lungs and lymph nodes of tumor-bearing mice. Infection of tumor cells by each attenuated *Lm* construct resulted in cell death by activation of NADPH oxidase and elevated levels of cytosolic reactive oxidative species (ROS). A more recent study by the same laboratory documented a similar tumor-homing ability of an attenuated *Lm* vector that expresses truncated LLO from an episomal plasmid (*Listeria*^at^) to pancreatic tumors in mice (Quispe-Tintaya et al., 2013). *Listeria*^at^ tropism was specific for primary tumors and metastatic lesions with very little accumulation in normal tissues three days after infection. In fact, *Listeria*^at^ appeared to be selective for the metastatic lesions of Panc-02 pancreatic tumor cells. To leverage this tumor tropism therapeutically, radioactively-labeled *Listeria*^at^ was administered to tumor-bearing mice. The radioactively-labeled *Listeria*^at^ markedly reduced the number of Panc-02 metastatic lesions in tumor-bearing mice demonstrating the potential of attenuated *Lm* constructs as tumor-targeting vectors (Quispe-Tintaya et al., 2013).

## *Lm* as an effective vaccine vector for tumor immunotherapy: preclinical studies targeting clinically relevant TAAs

*Lm*-based vaccines have been developed for numerous malignancies that demonstrate promising efficacy in preclinical models of cancer. Several of these vaccines, grouped by indication, are described in detail below and listed in Table 1.

**Table 1:** ***Lm*-based vaccines in development that target clinically-relevant tumor-associated antigens**.

**Target**	**Target antigen**	***Lm*-based Vaccine**	***Lm*-strain**	***Lm*-expressed Antigen**	**References**
Cervical cancer	HPV16 E7	*Lm*-E7	10403S (wt)	LLO signal sequence fused to HPV16 E7	Gunn et al., 2001
	HPV16 E7	*Lm*-LLO-E7 (ADXS-HPV)	XFL-7 (*prfA*-)	dtLLO fused to HPV16 E7	Gunn et al., 2001
	HPV16 E7	r*Lm*-E7	10403S (wt)	HPV16 E7 fused at the N-terminus with the LLO signal sequence and at the C-terminus with *E. coli* PhoA	Lin et al., 2002
	HPV16 E7	*Lm*-ActA-E7	XFL-7 (*prfA*-)	ActA a.a. 1-420 fused to HPV16 E7	Sewell et al., 2004a
	HPV16 E7	*Lm*-PEST-E7	XFL-7 (*prfA*-)	LLO a.a. 1-50 fused to HPV16 E7	Sewell et al., 2004b
	HPV16 E7	*Lm*-v1 and v2	*Lm*dd (*dal- dat*-)	dtLLO fused to HPV16 E7 expressed from a pCMV-driven plasmid delivered by *Lm*	Souders et al., 2006
	CRPV E1	E1-r*Lm*	10403S (wt)	CRPVE1 fused at the N-terminus with the LLO signal sequence and at the C-terminus with *E. coil* PhoA	Jensen et al., 1997
Breast cancer	Rat Her2/neu	*Lm*-LLO-EC1, EC2, EC3, IC1, and IC2	XFL-7 (*prfA*-)	dtLLO fused to selected regions of rat Her2/neu	Singh et al., 2005
	Human Her2/neu	*Lm*-hHer2/neu chimera	XFL-7 (*prfA*-)	dtLLO fused to chimeric protein containing epitopes from human Her2/neu	Seavey et al., 2009b
	Human Her2/neu	*Lm*-cHer2 (ADXS-cHER2)	*Lm*ddA (*dal- dat- actA*-)	dtLLO fused to chimeric protein containing epitopes from human Her2/neu	Shahabi et al., 2011
	Mouse ISG15	*Lm*-LLO-ISG15	XFL-7 (*prfA*-)	dtLLO fused to mouse ISG15	Wood et al., 2012
	Mouse MAGE-b	*Lm* LLO Mage-b_311-660_	XFL-7 (*prfA*-)	dtLLO fused to mouse Mage-b a.a. 311-600	Kim et al., 2008
	Human p53	*Lm*ddA-LLO-p53	*Lm*ddA (*dal- dat- actA*-)	dtLLO fused to human p53	Ishizaki et al., 2010
Tumor-associated vasculature	Mouse VEGFR-2 (Flk-1)	*Lm*-LLO-Flk-E1, E2, and l1	XFL-7 (*prfA*-)	dtLLO fused to selected regions of mouse VEGFR-2 (Flk-1)	Seavey et al., 2009a
	Human HMW-MAA	*Lm*-LLO-HMWMAA-C	XFL-7 (*prfA*-)	dtLLO fused to human HMW-MAA a.a. 2160-2258	Maciag et al., 2008
	Mouse CD105 (endoglin)	*Lm*-LLO-CD105A and B	XFL-7 (*prfA*-)	dtLLO fused to selected regions of mouse CD105 (endoglin)	Wood et al., 2011
Melanoma	Mouse TRP2, LCMV NP	*Lm*-TRP2-NP	10403S (wt)	Mouse TRP2 a.a. 24-191 fused at the N-terminus with the LLO signal sequence and at the C-terminus with LCMV NP a.a. 177-191 followed by *E. coli* PhoA	Bruhn et al., 2005
	Mouse TRP2	*Lm*-TRP2	10403S (wt)	Mouse TRP2 a.a. 24-191 fused at the N-terminus with the LLO signal sequence and at the C-terminus with *E. coli* PhoA	Bruhn et al., 2005
	Human HMW-MAA	*Lm*-LLO-HMWMAA-C	XFL-7 (*prfA*-)	dtLLO fused to human HMW-MAA a.a. 2160-2258	Maciag et al., 2008
Prostate cancer	Human PSA	*Lm*-LLO-PSA	XFL-7 (*prfA*-)	dtLLO fused to human PSA	Shahabi et al., 2008
	Human PSA	ADVX-31-142 (ADXS-PSA)	*Lm*ddA (*dal- dat- actA*-)	dtLLO fused to human PSA	Wallecha et al., 2009
Hepatocellular carcinoma	HBc, HBV-X, Human alpha-Fetoprotein, and Human MAGE-A	*Lm*-MPFG	*Lm*dd (*dal- dat*-)	dtLLO fused to a fusion peptide containing full-length HBc, HBx a.a. 52-60, HBx a.a 140-148, AFP a.a 158-166, MAGE a.a. 271-279 and a flag tag	Chen et al., 2012

### Cervical cancer

Early studies demonstrated the ability of *Lm*-based immunotherapies to induce therapeutically effective CTL responses against viral antigens in both an infection setting or when utilized as a model tumor antigen (Schafer et al., 1992; Ikonomidis et al., 1994, 1997; Pan et al., 1995a,b; Shen et al., 1995; Slifka et al., 1996; Jensen et al., 1997; Mata et al., 1998; Mata and Paterson, 1999). These studies provided the rationale to target *Lm*-based immunotherapies against virus-induced cancers. Cervical cancer is one of the most common forms of virus-induced cancer being the result of chronic infection with a transforming strain of human papillomavirus (HPV) (Cutts et al., 2007). It is especially prevalent among those with an active infection with a high-risk form of HPV, such as HPV16 or HPV18. HPV infection of mucosal epithelium tissue results in papilloma formation through the action of eight encoded proteins that are either expressed early in an infection to facilitate replication of HPV DNA with the E1, E2, E3, E4, E5, and E6 proteins or late in an infection to form the viral capsid with the L1 and L2 proteins. It is the early genes, E6 and E7 in particular, that allow for the papillomas to become malignant due to their inhibition of tumor suppressor genes, p53 and Rb respectively (Moody and Laimins, 2010). HPV E6 and E7 are constitutively expressed in the vast majority of cervical cancers caused by HPV infection due to their integration into the host genome and E7 is necessary for maintaining the malignant state of the tumor cells (Jabbar et al., 2012). It is for this reason that E6 and E7 make ideal targets for therapeutic strategies and why much of the effort to develop *Lm*-based immunotherapies for cervical cancer have focused specifically on targeting HPV E7.

#### HPV16 E7

The first study to apply recombinant *Lm* vaccine technology to a therapeutically relevant model of cancer in mice was performed by the Paterson laboratory and published in 2001 (Gunn et al., 2001). To develop their *Lm*-based therapeutics for cervical cancer, the group utilized a mouse model for HPV-induced malignancy, the TC.1 tumor model (Lin et al., 1996). The strategy for developing the first *Lm*-based immunotherapies was to engineer two separate recombinant *Lm* strains with each expressing HPV16 E7 due to its broad expression among all ano-genital malignancies (Gunn et al., 2001). However, one recombinant *Lm* expressed and secreted E7 alone, *Lm*-E7, while another strain was developed that expressed and secreted HPV16 E7 genetically fused to dtLLO, *Lm*-LLO-E7. After implanting TC.1 tumors in mice and allowing for establishment, mice were administered either vaccine and tumor growth monitored. While each E7-secreting vaccine successfully delayed tumor growth in comparison to untreated or control *Lm* treated mice, only *Lm*-LLO-E7 resulted in complete eradication of established tumors. Surprisingly, the increased anti-tumor efficacy of *Lm*-LLO-E7 in comparison to *Lm*-E7 was not the result of greater induction of E7-specific CTL responses in peripheral lymphoid organs as each vaccine was just as effective. Further studies determined that vaccination with *Lm*-E7 resulted in induction of greater numbers of CD4^+^ Tregs. In fact, it appeared that much of the reduced efficacy of *Lm*-E7 in comparison to *Lm*-LLO-E7 was due to Treg-mediated suppression of anti-tumor immune responses as Treg-depletion resulted in comparable anti-tumor responses. These results were confirmed in a subsequent study demonstrating that *Lm*-E7 preferentially induces Tregs that suppress effector cell function through production of IL-10 and TGF-β (Hussain and Paterson, 2004). Furthermore, work by Peng et al. found that *Lm*-LLO-E7 infection of BMDCs resulted in greater BMDC maturation, IL-2 production, and expansion of E7-specific CTLs than *Lm*-E7 infection (Peng et al., 2004). Lin et al provided additional evidence of the benefit of this approach in a study that reported significant anti-tumor protective immunity after vaccination with an *Lm*-based construct, r*Lm*-E7, that secreted E7 fused to both a portion of LLO and *E. coli* phoA (Lin et al., 2002). These studies demonstrate the promising therapeutic potential of *Lm*-based vaccines against cervical cancer but provide caution to others with the evidence that therapeutic vaccines may also induce peripheral tolerance if constructed improperly.

Previous studies have found that expression of HPV16 E7 can result in loss of responsiveness to IFN-γ (Park et al., 2000; Zhou et al., 2011). While this lack of responsiveness may be a resistance mechanism to limit the direct anti-viral and anti-tumor effect of IFN-γ, studies have suggested it may also impact anti-tumor immune responses (Beatty and Paterson, 2000, 2001). In fact, the initial study with *Lm*-LLO-E7 by Gunn et al. determined that the anti-tumor efficacy of *Lm*-LLO-E7 was dependent on the effects of IFN-γ (Gunn et al., 2001). To determine the role of IFN-γ in the anti-tumor response mediated by *Lm*-LLO-E7, Dominiecki et al utilized a TC.1 variant cell line unresponsive to IFN-γ due to constitutive expression of a dominant negative form of the IFN-γ receptor, termed mugR (Dominiecki et al., 2005). While TC.1-mugR tumors grew slightly slower than TC.1 parental tumors in the absence of therapy, they were resistant to the anti-tumor response mediated by *Lm*-LLO-E7. The mechanism for this resistance to *Lm*-LLO-E7 therapy appeared to correspond to the inability of CTLs to migrate into the tumor and effect tumor regression. Paterson and colleagues studied this phenotype further and discovered that IFN-γ stimulation of TC.1 tumor cells results in the production of a chemokine, CXCL9, that mediates infiltration of CD8^+^ T cells into TC.1 tumors after *Lm*-LLO-E7 vaccination (Guirnalda et al., 2013). Therefore, the effective anti-tumor immune response induced by *Lm*-LLO-E7 is due to a number of factors such as reduced peripheral tolerance and increased maturation of dendritic cells but this response is only effective if tumors remain compliant through responsiveness to IFN-γ, a determinant that may be useful to predict patient outcomes with *Lm*-based immunotherapies.

Numerous studies have found that responses to tumor immunotherapy are greatly enhanced when mechanisms maintaining peripheral tolerance are inhibited (Gunn et al., 2001; Duraiswamy et al., 2013; Mkrtichyan et al., 2013). While there are several pathways regulating peripheral tolerance, the PD-1 receptor pathway is gaining particular interest as a target for improving immunotherapies (Topalian et al., 2012). Interestingly, BMDCs infected with *Lm*-LLO-E7 were found to upregulate expression of the PD-1 ligand, PD-L1 (Peng et al., 2004; Mkrtichyan et al., 2013). Therefore, Khleif and colleagues sought to determine if activation of the PD-1 immune inhibitory pathway was limiting the efficacy of *Lm*-LLO-E7 (Mkrtichyan et al., 2013). Antibody-mediated blockade of PD-1 along with *Lm*-LLO-E7 did result in increased anti-tumor efficacy as determined by TC.1 tumor load, survival, and E7-specific T cell responses in comparison to vaccine alone. However, the *Lm*-LLO-E7 dose in this study was approximately 20-fold lower (5 × 10^6^ CFUs) than in previous studies (10^8^ CFUs) so it is unclear if this enhanced efficacy is only evident when given with a suboptimal dosage of *Lm*-LLO-E7 (Gunn et al., 2001; Guirnalda et al., 2013). Nevertheless, the results obtained from this study could lead to a very effective synergistic clinical treatment regimen especially with the promising results of both *Lm*-LLO-E7 and anti-PD-1 treatment separately in clinical trials (Maciag et al., 2009; Petit and Basu, 2013; Topalian et al., 2012).

### Breast cancer

#### Her2/neu

After finding preclinical success treating models of virus-induced cancer, development progressed on *Lm*-based vaccines for other challenging indications such as breast cancer where the only identified targets are self-TAAs, such as Her2/neu. Her2 is a epidermal growth factor receptor family protein that is overexpressed in roughly 25% of human breast tumors and correlates with poor prognosis (Slamon et al., 1987). While most vaccines targeting Her2 were primarily used in prophylactic settings, Singh et al rationalized that the use of *Lm* as a vaccine vector may induce a robust CTL response that is therapeutically effective (Singh et al., 2005). However, in order to construct *Lm*-based vaccines against a large membrane-bound receptor like Her2, several constructs were required to be developed. Each construct contained a portion of the intracellular (*Lm*-LLO-IC1 and *Lm*-LLO-IC2) or the extracellular (*Lm*-LLO-EC1, *Lm*-LLO-EC2 and *Lm*-LLO-EC3) portion of the full-length rat Her2/neu protein to allow for efficient production and secretion by *Lm*. Therapeutic vaccination with each of the constructs individually resulted in reduced or static growth of NT-2 breast tumors, a tumor cell that constitutively expresses rat Her2/neu under the mouse mammary tumor virus (MMTV) promoter (Singh et al., 2005). In comparison to DNA vaccines targeting Her2, *Lm*-based vaccines demonstrated significantly greater therapeutic efficacy. Interestingly, the efficacy of *Lm*-based Her2 vaccines was not due solely to the bacterium as a DNA vaccine study suggested the fusion of the Her2 antigenic regions to dtLLO is also an important factor (Singh and Paterson, 2006b). The mechanism for this improved efficacy of *Lm* or LLO-based Her2 vaccines appears to be in their ability to induce a greater repertoire of Her2-specific CTLs. In fact, vaccination with *Lm*-based Her2 vaccines also revealed more epitopes than had been previously identified with a DNA vaccination strategy (Singh et al., 2005). This suggests that vaccination against Her2 with *Lm* or LLO-based vaccines generated both a quantitatively and qualitatively different response than by other methods and with greater therapeutic efficacy.

While initial studies with the *Lm*-Her2 vaccines demonstrated promising efficacy against a mouse tumor cell line that expresses rat Her2/neu, they were performed in a mouse model that was not fully tolerant to the antigen. Although rat and mouse Her2/neu are roughly 94% homologous, rat Her2/neu is immunogenic in mice (Nagata et al., 1997). Therefore, future studies were performed in an MMTV-Her2/neu-transgenic mouse model that constitutively expresses rat Her2/neu under control of the MMTV promoter and shows profound tolerance to rat Her2/neu (Guy et al., 1992; Reilly et al., 2000). Although fewer mice responded to *Lm*-Her2 therapy, complete tumor regressions were observed. Further studies elucidated several mechanisms for the vaccines not reaching their full therapeutic potential. First, after vaccination with *Lm*-Her2 vaccines, tumors would undergo immunoediting, a process wherein immunological pressure against a TAA will result in selective outgrowth of tumor cells that have mutations in the targeted antigen (Singh and Paterson, 2007a). Second, central tolerance to self-TAAs, in particular for these studies with Her2, is detrimental to the induction of high avidity tumor-specific CTLs (Singh and Paterson, 2007b). When FVB/N Her2/neu transgenic mice bearing Her2-positive tumors were vaccinated with *Lm*-based Her2 vaccines, CTLs were generated against previously revealed epitopes but the avidity of those CTLs was reduced by at least one log in comparison to CTLs generated in a wild-type mouse. Finally, peripheral tolerance mediated by suppressive cell types such as Tregs also limits the efficacy of *Lm*-based tumor immunotherapies (Singh and Paterson, 2007b). Antibody-mediated depletion of Tregs concomitant with *Lm*-based vaccination against Her2 resulted in enhanced anti-tumor efficacy, therefore, providing a possible therapeutic avenue for improved efficacy in the clinic (Singh and Paterson, 2007b).

**Figure 3:**
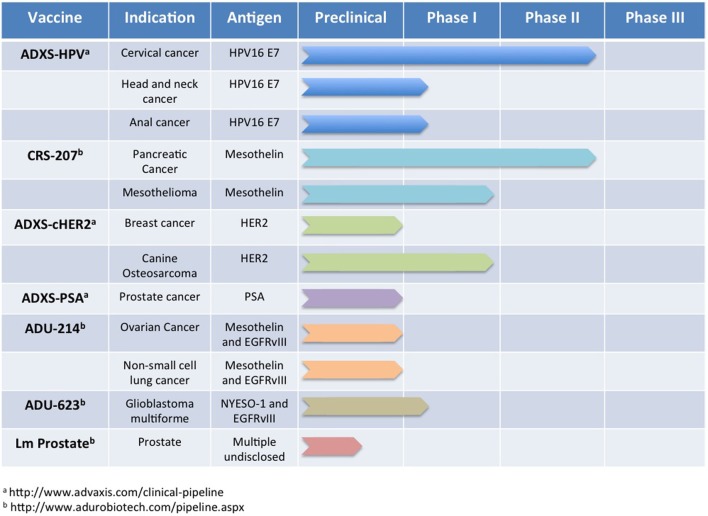
***Lm*-based vaccine clinical trials pipeline**. Numerous *Lm*-based vaccines have progressed through the discovery and preclinical phase of development and are now being administered to oncology patients for a number of indications. In this figure, we summarize publicly available information for some of the *Lm*-based vaccines in clinical testing currently or planned for clinical testing in the near future.

To translate the success of *Lm*-based immunotherapies targeting rat Her2/neu into the clinic, development of several vaccines was undertaken that expressed regions or defined epitopes within human Her2 (Seavey et al., 2009b; Shahabi et al., 2011). As in the previous studies with rat Her2/neu, *Lm*-based vaccines were developed that expressed separate regions of the intracellular (*Lm*-LLO-hIC1) and extracellular (*Lm*-LLO-hEC1 and *Lm*-LLO-hEC2) portions of human Her2. Due to the high degree of homology between human and rodent Her2/neu and the presence of defined H-2^q^ epitopes within the portions from human Her2 (Seavey et al., 2009b), mouse breast tumor models such as NT-2, 4T1, and the MMTV-Her2/neu transgenic could be used to assess efficacy. Each of these vaccines was found to be equally as effective at inducing Her2/neu-specific CTL responses and controlling tumor growth in each of the tumor models as the previous rat Her2/neu-based constructs. However, to improve the breadth of the immune response against Her2, an additional vaccine was developed that contained most of the HLA epitopes found within *Lm*-LLO-hIC1, *Lm*-LLO-hEC1, and *Lm*-LLO-hEC2 but expressed as a single polyvalent antigen (Seavey et al., 2009b). This chimeric Her2 vaccine, named *Lm*-hHer2/neu chimera, demonstrated significant anti-tumor efficacy and was able to delay autochthonous tumor formation in a tolerized Her2/neu transgenic mouse. Additionally, the *Lm*-hHer2/neu chimera was able to prevent lung metastasis formation from intravenous challenge with the highly aggressive 4T1-Luc breast tumor model. This impressive efficacy of *Lm*-hHer2/new chimera at eliciting a potent anti-tumor response was highlighted by the relatively low expression of Her2/neu by 4T1-Luc tumor cells (Seavey et al., 2009b). The success of the *Lm*-hHer2/neu chimera vaccine resulted in its further development into a new *Lm*-based vector that is absent of any antibiotic resistance genes, and is therefore more easily translatable to the clinic (Shahabi et al., 2011). This new vaccine, ADXS-cHER2, demonstrated improved efficacy over *Lm*-hHer2/neu chimera in autochthonous Her2/neu breast tumor studies but also an impressive ability to protect mice from intracranial challenge from the EMT6-Luc breast tumor cell line. The preclinical development of *Lm*-based chimeric Her2 vaccines predicts a promising future that will be determined in clinical trials that are ongoing in canines and currently planned for humans (Figure 3).

#### Interferon stimulated gene 15 (ISG15)

While Her2 is a promising target for breast cancer tumor immunotherapy, its wide application is limited since only about 25% of breast cancer patients have elevated expression of Her2 in their tumors (Slamon et al., 1987). This is especially problematic for breast cancer patients with tumors that lack expression of Her2/neu, progesterone receptor, and the estrogen receptor, denoted as triple-negative, and have few therapeutic options available (Hudis and Gianni, 2011). Therefore, the discovery of novel TAAs for use in breast tumor immunotherapy is a desired goal. Progress in this area of research was recently reported in a study that targeted a small ubiquitin-like protein elevated in even triple-negative breast tumors, ISG15, with an *Lm*-based immunotherapy (Bektas et al., 2008; Wood et al., 2012). After validating overexpression of ISG15 in mouse models of breast cancer and developing an *Lm*-based vaccine that expresses ISG15 fused to dtLLO, Wood et al found that vaccination of mice with *Lm*-LLO-ISG15 resulted in induction of ISG15-specific CTL responses. In a therapeutic setting, treatment with *Lm-LLO-ISG15* significantly reduced the growth of breast tumors and the number of metastatic lesions in the lungs of tumor-bearing mice. When administered prophylactically in MMTV-Her2/neu transgenic mice, autochthonous mammary tumor formation was significantly delayed as compared to a control *Lm*. These results suggest that ISG15 is not only a novel immunotherapeutic target for breast cancer but also that *Lm*-based vaccines are powerful platforms to validate TAA discovery.

#### Melanoma-associated antigen (MAGE)-b

MAGE are a type of cancer/testis antigens commonly targeted in tumor immunotherapy due to their limited expression in normal tissues (Simpson et al., 2005). MAGE-b, in particular, is highly expressed in over 90% of breast tumors (De Backer et al., 1995; Park et al., 2002). For this reason, Kim et al constructed several *Lm*-based vaccines containing regions of MAGE-b fused to dtLLO (Kim et al., 2008). In a mixed prophylactic and therapeutic vaccination regimen, mice were administered twice with one of four *Lm*-Mage-b vaccines prior to tumor challenge with 4T1 mammary carcinoma cells and then subsequent administration of a third vaccination. Surprisingly, vaccination with one vaccine, *Lm*-LLO-Mage-b/2nd resulted in significantly reduced 4T1 lung metastases as compared to control vaccination but no impact on primary tumor growth. Further examination discovered that elevated expression of IL-6 by the primary tumor was the likely culprit for this anti-tumor effect being limited to only metastatic disease. An additional mechanism of *Lm*-LLO-Mage-b/2nd anti-tumor efficacy was subsequently discovered involving direct killing of infected tumor cells though elevation of cytosolic ROS, as discussed above (Kim et al., 2009).

#### p53

The tumor suppressor p53 is commonly mutated in tumors and correlates with decreased survival in breast cancer (Harris and Hollstein, 1993). However, mutations in p53 can also result in tumor cell-specific overexpression and render them susceptible to killing by p53-specific CTLs (Zambetti and Levine, 1993; Nikitina et al., 2001). Therefore, Ishizaki et al. constructed an *Lm*-based vaccine targeting human p53, *LmddA*-LLO-p53, and assessed its anti-tumor efficacy against 4T1 tumor cells stably expressing mutant human p53 (4T1p53) in tolerant human p53 knock-in (Hupki) mice (Ishizaki et al., 2010). While *LmddA*-LLO-p53 administration alone provided some prophylactic and therapeutic efficacy against 4T1p53 tumors, p53-specific immunity and prophylactic efficacy were dramatically improved when given with a modified vaccinia Ankara vaccine expressing human p53 (MVA-p53) in a heterologous prime/boost approach. Therapeutic efficacy and survival were also further improved when adjuvants (Poly I:C and CpG-ODN) were incorporated into the heterologous prime/boost regimen. Therefore, this study provides additional evidence that maximal anti-tumor efficacy of *Lm*-based vaccines may sometimes be achieved only when given in a combinatorial approach (Hannan et al., 2012; Mkrtichyan et al., 2013).

### Tumor-associated vasculature

*Lm*-based vaccines targeting TAAs have demonstrated promise in numerous preclinical studies, however, the appearance of tumor immunoediting and tumor immune escape, likely due in part to the genetic instability of tumor cells (Cahill et al., 1998), has presented a challenge (Singh and Paterson, 2007a). Therefore, focus turned to targeting TAAs expressed by cell types believed to have greater genetic stability such as the tumor-associated vascular endothelial cells (Seavey and Paterson, 2009; Wood et al., 2011). Tumor-associated vascular endothelial cells have been successfully targeted with passive immunity to treat cancer for several years but a CTL-mediated approach is still lacking (Kim et al., 1993). Currently, there are several antigens associated with tumor vascular endothelial cells with the most well characterized being the vascular endothelial growth factor (VEGF) and it's receptor (VEGFR) but others such as Cluster of Differentiation 105 (CD105) are also proving to be promising targets for *Lm*-based tumor immunotherapy (Goel and Mercurio, 2013).

#### High molecular weight melanoma-associated antigen (HMW-MAA)

HMW-MAA was initially discovered as an antigen highly expressed in melanomas and other malignancies derived from the neural crest and a previous target of tumor immunotherapy (Campoli et al., 2004; Chang et al., 2004). In an effort to determine if an *Lm*-based immunotherapy targeting HMW-MAA would be effective against mouse models of melanoma, initial studies were performed against the B16F10 melanoma cell line stably expressing HMW-MAA along with an *Lm* vaccine expressing a region of HMW-MAA, *Lm*-LLO-HMWMAA-C (Maciag et al., 2008). While *Lm*-LLO-HMWMAA-C successfully reduced growth of a melanoma tumor expressing HMW-MAA, unexpected results were found in experiments targeting tumor cells that did not express HMW-MAA. Most strikingly, in two breast cancer models, *Lm*-LLO-HMWMAA-C, reduced breast tumor burden and increased time to progression in a Her2/neu transgenic autochthonous breast tumor model. Further examination of this unexpected efficacy determined that expression of the murine HMW-MAA homolog, AN2, was highly expressed by pericytes in the tumor vasculature. Pericytes are mesenchymal cells that express AN2 and are associated with, and stabilize, vasculature in tumors and the periphery (Schneider et al., 2001; Baluk et al., 2005; von Tell et al., 2006). After *Lm*-LLO-HMWMAA-C vaccination, CD8^+^ T cells colocalized with the tumor vasculature and the number of pericytes associated with the tumor vasculature was significantly decreased. Importantly, targeting of pericytes with *Lm*-LLO-HMWMAA-C did not impact normal processes associated with neovascularization such as wound healing and pregnancy. These observations may suggest that the specific susceptibility of tumors to pericyte-targeting therapy is due to the already decreased numbers of pericytes associated with tumor vasculature (Eberhard et al., 2000).

#### VEGFR2/Flk-1

Folkman's hypothesis that limiting tumor growth could be achieved by targeting tumor angiogenesis in order to limit the necessary oxygen and nutrient supply has been realized with the introduction of bevacuzimab, an antibody against VEGF, into the clinic (Hurwitz et al., 2004). VEGF receptors are found on activated vascular endothelium and the murine homolog of VEGFR2, Flk-1, plays an important role in tumor neovascularization and growth (Kim et al., 1993; Millauer et al., 1993, 1994). However, successful passive immunotherapies such as bevacuzimab have been met with therapeutic resistance (Ellis and Hicklin, 2008). In order to determine the efficacy of an active CTL-mediated immune responses against Flk-1 in tumor immunotherapy, three separate *Lm*-based vaccines were constructed based on regions of Flk-1 with vaccines targeting extracellular portions of the protein, *Lm*-Flk-E1 and *Lm*-Flk-E2, and intracellular, *Lm*-Flk-I1 (Seavey et al., 2009a). While each vaccine was able to induce specific IFN-γ responses against an MHC Class I epitope within their respective regions, only two of the vaccines, *Lm*-FlkE1 and *Lm*-FlkI1, were able to significantly inhibit Her2/neu+ breast tumor growth, reduce tumor vascularization, and induce secondary T cell responses against the non-targeted TAAs, a process known as epitope spreading (Vanderlugt and Miller, 2002). Interestingly, the anti-tumor efficacy of the Flk-1 targeting vaccines was completely abrogated when examined in a tolerant Her2/neu transgenic mouse. These results support a hypothesis that CTL-mediated targeting of antigens on activated vascular endothelium, such as VEGFR2, can result in anti-tumor efficacy but epitope spreading to additional TAAs is a requirement for this efficacy. Support for this theory was found in a follow-up study demonstrating that autochthonous tumor formation in Her2/neu transgenic mouse after *Lm*-Flk-E1 or *Lm*-Flk-I1 vaccination correlated with the occurrence of mutations in Her2/neu CTL epitopes within those tumors (Seavey and Paterson, 2009). Therefore, these studies suggest that therapeutic vaccines targeting antigens associated with tumor vasculature alone may not reach their full potential unless tolerance to TAAs is overcome. This predicts a possible promising therapeutic strategy of pairing one *Lm*-based vaccine targeting tumor vasculature with another vaccine that is able to break central tolerance to a TAA such as *Lm*-cHer2 to maximize therapeutic efficacy.

#### Endoglin/CD105

While previous studies proved Flk-1 to be an effective target for impacting tumor vasculature, further studies sought to validate additional promising tumor vasculature targets in an *Lm*-based immunotherapy approach. One subsequent study focused on a member of the TGF-β receptor complex, endoglin (CD105), which is highly expressed on the vascular endothelium of tumors and believed to play a role in tumor neovascularization (Wang et al., 1993; Perez-Gomez et al., 2005). Previous studies targeting CD105 with an antibody had already met with some success at controlling tumor burden in a clinical trial and preclinical models of cancer (Matsuno et al., 1999; Rosen et al., 2012). To determine the efficacy of targeting CD105 with an active immunotherapy approach, two *Lm*-based vaccines were engineered to secrete different regions of CD105 fused to dtLLO (Wood et al., 2011). Each vaccine, *Lm*-LLO-CD105A and *Lm*-LLO-CD105B, were found to effectively reduce the growth of Her2/neu+ breast tumors and delay progression of autochthonous tumor progression in a Her2/neu transgenic mouse. In addition, vaccination against CD105 reduced lung metastases in the 4T1 mouse model of metastatic breast cancer and led to epitope spreading to TAAs as seen previously with the Flk-1 vaccines. Tumor-associated expression of CD31 and hemoglobin were also reduced after CD105 vaccination demonstrating its promise as a target for CTL-mediated approaches to limit tumor angiogenesis.

### Prostate cancer

#### Prostate specific antigen (PSA)

The current standard of care for prostate cancer patients involving radiation and chemotherapy still does not prevent roughly 10–30% of men from having recurrent malignancy (Katz and McKiernan, 2007). Provenge administration in these patients results in successful but limited extension of lifespan but it carries with it the difficulties and high cost associated with autologous cell-based vaccines (Simoens, 2012). While Provenge targets PAP, improved efficacy may be found with another promising target for prostate tumor immunotherapy, prostate-specific antigen (PSA), a protein widely expressed in prostate adenocarcinomas (Cunha et al., 2006). PSA-specific CTL-mediated approaches developed to therapeutically treat prostate cancer have shown efficacy in the clinic and provided the rationale for development of an *Lm*-based vaccine targeting PSA, *Lm*-LLO-PSA (Heiser et al., 2002; Shahabi et al., 2008). *Lm*-LLO-PSA vaccination in mice was successful at generating PSA-specific CTL responses. Administration of *Lm*-LLO-PSA to mice bearing T-PSA23 prostate tumors, TRAMP-C murine prostate adenocarcinoma tumors stably expressing PSA, resulted in eradication of over 80% of tumors along with reduced infiltration of Tregs into tumors as compared to a control vaccine (Shahabi et al., 2008). In a comparison of vaccine vectors, *Lm*-LLO-PSA was also found to be the most effective at reducing prostate tumor burden in mice as compared to DNA and vaccinia virus-based vaccines expressing the same antigen.

A follow-up study with an updated *Lm*-based vaccine targeting PSA, but devoid of antibiotic selection markers, ADXS31-142 (Wallecha et al., 2009) was recently undertaken to determine whether its anti-tumor efficacy could synergize with the radiation therapy that is commonly administered to prostate cancer patients (Wallecha et al., 2009; Hannan et al., 2012). After implantation of T-PSA23 tumors, mice were treated with either radiation followed by three doses of ADXS31-142, radiation alone, or ADXS31-142 alone. Each treatment resulted in significantly lower tumor burden as compared to control *Lm* vaccination but radiation followed by ADXS31-142 vaccination resulted in synergistic therapeutic anti-tumor efficacy. The mechanism for this synergistic application of radiation and *Lm* vaccine appeared to be due to a robust four-fold increase in PSA-specific CTLs in the spleens of treated mice. These results are in line with previous studies that documented synergism of irradiation along with tumor immunotherapy and provide a rational approach for administration of *Lm*-based vaccines in prostate cancer patients (Kantoff et al., 2010b).

### Hepatocellular carcinoma

#### Multiple peptide fusing genes (MPFG)

In order to mitigate the emergence of tumor immune escape due to mutation of a single targeted TAA epitope, some have developed chimeric *Lm*-based vaccines that incorporate multiple epitopes from regions spanning an entire TAA (Seavey et al., 2009b; Shahabi et al., 2011). While these studies have met with success, loss of complete TAA expression due to immune pressure, as previously observed with Her-2/neu, may render even this approach ineffective (Kmieciak et al., 2007). Therefore, to further impede the emergence of tumor immune escape, others have developed polyvalent therapeutic tumor vaccines that incorporate epitopes from several TAAs (Tanaka et al., 2002). Early *Lm*-based polyvalent vaccines have either contained model tumor antigens (Bruhn et al., 2005) or were not validated for *in vivo* efficacy (Sinnathamby et al., 2009) but a recent study by Chen et al. documents the validation of an *Lm*-based polyvalent vaccine targeting clinically relevant TAAs in a model of hepatocellular carcinoma (HCC) (Tanaka et al., 2002; Chen et al., 2012). HCC is a prevalent cause of cancer-related deaths worldwide, especially in countries such as China where oncogenic Hepatitis B virus (HBV) infection is endemic (Chen et al., 2012). In order to develop an effective *Lm*-based therapeutic vaccine to treat HCC that overcomes the challenges of immune escape of previous immunotherapies, the authors constructed a vaccine that expressed the entire HBV core protein (HBc) along with validated HLA-A2 epitopes for additional TAAs including HBV-X protein, alpha-fetoprotein, and MAGE-A. The resulting fusion protein was named multiple peptide fusing genes (MPFG) and the *Lm*-based vaccine that expressed this fusion protein, *Lm-MPFG. Lm-MPGF* administration to HLA-A2 transgenic mice induced robust IFN-γ and cytolytic responses against the MPFG antigens. When mice were vaccinated prophylactically or therapeutically after inoculation with Hepa1-6 tumor cells expressing MPFG and HLA-A2, *Lm*-MPFG treatment led to reduced tumor burden and increased tumor-free survival in comparison to a control *Lm*. The anti-tumor efficacy of *Lm*-MPFG correlated with increased infiltrations of MPFG-specific CD8^+^ T cells and increased cytolytic potential of CTLs. Additionally, prophylactic and therapeutic administration of *Lm*-MPFG resulted in significantly reduced number of Tregs in tumors and reduced production of anti-inflammatory cytokines such as TGF-β and IL-10 by the tumor-infiltrating Tregs. This study provides the first evidence that *Lm*-based immunotherapies could be effective in the treatment of HCC but it also demonstrates the validation of a polyvalent *Lm*-based vaccine approach that will prove useful in limiting tumor immune escape.

### Melanoma

#### TRP-2

Early studies with Lm-based tumor immunotherapies targeting a mouse model of melanoma, B16F10-NP, demonstrated their potency against this malignancy. However, these studies were targeting the model tumor antigen influenza nucleoprotein, NP, and not a melanoma-specific TAA that is clinically relevant (Pan et al., 1999). One melanoma-specific TAA that previously was found to be immunogenic and a validated target for tumor immunotherapy is Tyrosinase-related protein-2 (TRP-2) (Bloom et al., 1997). TRP-2 is a melanogenic enzyme expressed specifically by melanocytes and melanoma cells (Wang et al., 1996). To determine the therapeutic efficacy of targeting TRP-2 with Lm-based immunotherapy, Bruhn et al. constructed several Lm-based vaccines expressing either TRP-2 alone or fused to an MHC class I H-2D^b^-restricted epitope from LCMV NP (a.a. 396–404) (Bruhn et al., 2005). While either vaccine was capable of inducing TRP-2-specific CD8+ T cell responses, studies determining anti-tumor efficacy were subsequently carried out with only the Lm-TRP2-NP construct. After two prophylactic vaccinations with Lm-TRP2-NP and subsequent challenge with B16 tumors, mice receiving the TRP-2 vaccine had significantly reduced tumor burden as compared to mice that received the control Lm, Lm-NP, or empty Lm vector. The Lm-TRP2-NP vaccine was also found to be therapeutically effective against B16-MO5 metastatic lesions, a variant of the B16 tumor cell line stably expressing chicken ovalbumin (Falo et al., 1995). A follow-up study also demonstrated the ability of Lm-TRP2-NP to provide protective immunity in a B16 brain metastasis model (Prins et al., 2006). To further improve the anti-tumor efficacy of Lm-TRP2-NP, it was administered in combination with the TLR7 agonist, imiquimod (Craft et al., 2005). Imiquimod was previously found to be a strong adjuvant and lead to reduced tumor growth alone. When administered along with Lm-TRP2-NP, it enhanced the prophylactic anti-tumor efficacy of Lm-TRP2-NP in both primary and metastatic B16 tumor challenge models. The findings from these studies validate Lm-based immunotherapies as effective inducers of anti-tumor immune responses to sites of metastatic spread that are normally considered immune-privileged, a finding later confirmed in a mouse model for breast cancer by Shahabi et al. (2011).

## Clinical trials with *Lm*-based vaccines

### First safety trial with empty *Lm* vaccine strain

The first human safety trial for an attenuated *Lm*-based empty vaccine vector was reported in 2002 by Angelakopoulos et al. (2002). For this study, the authors sought to administer an *Lm* strain that was highly attenuated with little risk of reversion to a more virulent state. To accomplish this, the *Lm* strain used for this study contained genetic deletions of two common virulence factors produced by *Lm*, *actA*, and *plcB*. Additional measures to confirm the safety of this *Lm* vaccine strain confirmed the inability of this strain to revert to a more virulent phenotype after 30 serial passages on agar. Twenty healthy volunteers ingested, in 25 mL of saline, the *Lm* vaccine strain in escalating doses from 10^6^ CFUs up to 10^9^ CFUs. While 75% of the volunteers had detectable shedding of the *Lm* vaccine strain in their stool for up to four days after administration, no serious adverse events occurred with only transient increases in liver enzymes observed in two volunteers. Antibody responses to the *Lm* vaccine strain were minimal with no detectable *Lm*-specific IgA, but all four volunteers in the group receiving the highest dose (10^9^ CFU) had detectable *Lm*-specific IFN-γ responses suggesting the vaccine was successful at inducing the formation of *Lm*-specific T cell responses. The results from this first human trial with an *Lm* vaccine strain provided confidence in the ability of attenuated *Lm* strains to safely and effectively induce antigen-specific T cell responses in humans.

### *Lm*-LLO-E7 (ADXS-HPV) for cervical cancer

The preclinical success of *Lm*-LLO-E7 resulted in its introduction into the clinic for a Phase I trial in patients with invasive carcinoma of the cervix (ICC), a prevalent HPV-induced cancer in many developing countries (Cutts et al., 2007; Maciag et al., 2009). As a Phase I trial, the safety and tolerability of *Lm*-LLO-E7 in humans were of primary interest but additional parameters such as objective response as determined by Response Evaluation Criteria in Solid Tumors (RECIST) criteria and overall survival were also measured (Eisenhauer et al., 2009). Fifteen patients with ICC, most having failed previous therapeutic regimens, were placed into three treatment groups with each scheduled to receive two vaccinations three weeks apart with 1 × 10^9^, 3.3 × 10^9^, or 1 × 10^10^ CFUs of *Lm*-LLO-E7. All patients reported adverse events after receiving *Lm*-LLO-E7 with the most common being pyrexia in all patients following by vomiting, headache, and anemia. All adverse events were transient and grade 3 or lower with the most severe related to *Lm*-LLO-E7 infection. Dose-limiting toxicities were observed, however, only in the group receiving the highest dose of 1 × 10^10^ CFUs. In addition, a delayed increase in liver enzymes was observed in some patients one week after administration. This increase was likely not due to a current infection as all patients received antibiotics three days after administration of *Lm*-LLO-E7. While two deaths occurred, unrelated to *Lm*-LLO-E7 administration, over half of the remaining subjects had objective stable disease by RECIST criteria with one patient having a partial response. Overall survival for all evaluable patients was 347 days at the end of the study with two surviving subjects. This compares favorably to the current median overall survival of ICC patients receiving standard of care chemotherapy ranging from 6.5 to 9.4 months (Monk et al., 2009). Therefore, the results from this study were very promising in terms of tolerability and overall survival in a patient population with very few effective therapeutic options. While the adverse events observed in this study were of a higher grade and more prevalent than those observed in the previous Angelakopoulos et al. study (2002), the intravenous administration in this study as compared to oral administration likely accounts for the increased virulence of the infection.

The promising results from the Phase I trial with *Lm*-LLO- E7 (Maciag et al., 2009) resulted in its further development to a Phase II clinical trial in India initiated in 2010 and ended with its last patient visit in the fall of 2013 (Petit and Basu, 2013). The study enrolled 110 patients with ICC and placed them into two treatment groups, one receiving three doses of 1 × 10^9^ CFU of *Lm*-LLO-E7 and the second receiving 4 doses of 1 × 10^9^ CFU of *Lm*-LLO-E7 along with cisplatin, an alkylating platinum-based chemotherapy (Siddik, 2003), The final 12-month survival among all patients was 36% and 18-month survival is currently at 28% at study end. In addition, five complete responses and six partial responses were observed. These results compare favorably with the previous Gynecologic Oncology Group (GOG) trials featuring a combined paclitaxel and cisplatin treatment regimen with a 12-month survival of 32% and 18-month survival at 22%. There were no observed differences in responses or survival between the two treatment arms. Although Lm-LLO-E7 was constructed using HPV16 E7, responses were observed in patients with tumors transformed by several different high risk HPV strains including HPV16, 18, 31, 33, and 45.

Mild to moderate adverse events were observed in less than half of patients with only 2% experiencing grade 3 or greater adverse events. This also compares favorably to the current standard-of-care chemotherapy with grade 3 or greater adverse event rates nearing 100% (Monk et al., 2009). The adverse events related to *Lm*-LLO-E7 appeared to correlate with the magnitude of innate immunity activation after administration suggesting serum cytokines could be used in the future to monitor patient responses. Overall, the combined results of each of the *Lm*-LLO-E7 clinical trials demonstrate an effective new therapeutic option for patients battling advanced cervical cancer but with reduced side-effects compared to current treatments (Monk et al., 2009). The promising results from the completed *Lm*-LLO-E7 clinical trails have resulted in its continued development as a therapeutic for advanced cervical cancer, with another Phase II study currently being conducted by the Gynecologic Group in the US, but also other HPV-associated malignancies. These studies were performed by Advaxis Inc. who have renamed the *Lm*-LLO-E7 vector, ADXS-HPV (http://www.advaxis.com/clinical-pipeline).

### ANZ100 and CRS207

A pair of studies reported by Le et al. (2012) described the administration two novel live attenuated double-deletion (LADD) *Lm* vaccine strains (Le et al., 2012). The first study, much like Angelakopoulos et al. (2002), involved administration of an empty *Lm* vaccine strain, ANZ-100, that contains deletions for two *Lm* virulence factors, *actA*, and *inlB*. The ANZ-100 study consisted of 9 patients (6 with colorectal cancer, 2 with pancreatic ductal carcinoma, and 1 with melanoma) that each received a single intravenous infusion of either 1x10^6^, 3x10^7^, or 3x10^8^ CFUs of the attenuated *Lm*. ANZ-100 was well tolerated by the patients at all doses with transient adverse events such as lymphopenia, hyperglycemia, hypophosphatemia, and fever in most patients but no observed dose-limiting toxicities. Within 2 days after dosing, a sharp drop in circulating NK cells and increased NK cell expression of CD38 suggested that ANZ-100 was stimulating NK cell activation and extravasation into infected tissues. ANZ-100-induced NK cell activation corresponded with a sharp transient rise in serum proinflammatory cytokines and chemokines in a dose-dependent manner. While patient follow-ups were not reported, this study demonstrated that LADD are safe for use in cancer patients and can induce proinflammatory responses that are critical for effective anti-tumor immunity.

Further work continued with a similar LADD construct but one that expressed the TAA mesothelin as part of a fusion protein with ActA, termed CRS-207. Mesothelin is a glycoprotein normally expressed in mesothelial cells but highly overexpressed in a number of different tumor types making it a promising target for tumor immunotherapy (Thomas and Hassan, 2012). The CRS-207 study was comprised of 17 patients, each with a type of cancer associated with elevated expression of mesothelin including ovarian, pancreatic, mesothelioma, and non-small cell lung cancer. Each patient received four infusions of CRS-207 three weeks apart and separated into four groups based on the dosage of CRS-207 received, either 10^8^, 3 × 10^8^, 10^9^, or 10^10^ CFUs. Treatment-related toxicities were comparable to the ANZ-100 study with one observed dose-limiting toxicity in a patient receiving 10^10^ CFUs. The maximum-tolerated dose was therefore determined to be 10^9^ CFU of CRS-207. As previously observed with ANZ-100, CRS-207 also induced proinflammatory cytokines and chemokines but the dose-dependence was not as clear with the highest dose, 10^10^ CFUs, inducing the least amount of proinflammatory cytokines such as IFN-γ and IL-12. As only one patient received the highest dose, the lack of dose-dependence could be due to lack of statistical power. Mesothelin and LLO-specific T cell responses were observed in patients from all groups receiving up to 10^9^ CFUs of CRS-207. In terms of efficacy, over 37% of patients survived for greater than 15 months with responses and survival independent of the dosage administered and the type of cancer treated. Interestingly, the best predictor of long-term survival in this population after CRS-207 administration was the presence of LLO-specific T cell responses in the periphery but not those to mesothelin. The authors suggest this correlation may be predictive of the immune competency of the patient. The results of the CRS-207 Phase I study were promising in terms of overall survival for a set of patients with further work needed to elucidate the mechanisms governing its efficacy.

## *Lm*-based immunotherapies going forward

The current state of *Lm*-based immunotherapies provides a great deal of optimism for their future in the clinic as safer and more effective therapeutic options than current treatment regimens as evidenced by the recent clinical findings with ADXS-HPV and a robust clinical pipeline summarized in Figure 3 (Maciag et al., 2009; Petit and Basu, 2013). However, several preclinical studies covered within this review suggest advancements that will likely influence construction strategies going forward and lead to improved *Lm*-based immunotherapies. As the studies with *Lm*-Her2 constructs demonstrated, one major challenge for tumor immunotherapies targeting a single TAA is the eventual selection for mutated TAAs in the targeted tumors and immune escape (Singh and Paterson, 2007a). The study by Chen et al, however, suggests that incorporating multiple epitopes from several antigens into a polyvalent vaccine could be an effective strategy going forward for *Lm*-based immunotherapies to mitigate immune escape (Chen et al., 2012). In addition to the development of polyvalent *Lm*-based vaccines, it is expected that new constructs may leverage the tumor tropism of their attenuated *Lm* vaccines (Kim et al., 2009; Quispe-Tintaya et al., 2013). As elegant studies from the Gravekamp laboratory have elucidated, the primary and metastatic tumor tropism and killing by *Listeria* could possibly be harnessed to effectively treat stubborn malignancies such as pancreatic ductal carcinoma (Quispe-Tintaya et al., 2013). This tumor tropism suggests a possible synergistic strategy of *Lm*-based vaccines going forward to deliver therapeutic proteins or expression plasmids to the tumor microenvironment as previously demonstrated (Stritzker et al., 2008) in addition to TAAs for induction of a tumor-specific CTL response (Souders et al., 2006; Stritzker et al., 2008). Finally, the synergism of *Lm*-based immunotherapies in combination with radiation, adjuvants, and therapeutic antibodies suggests a versatility that we are only just uncovering (Ishizaki et al., 2010; Hannan et al., 2012; Mkrtichyan et al., 2013). Each of these promising developments along with growing list of ongoing and upcoming clinical trials of *Lm*-based immunotherapies for cancer demonstrate that we are just now realizing the goals of William Coley a century ago.

### Conflict of interest statement

Yvonne Paterson has a financial interest in Advaxis, Inc., a vaccine and therapeutic company that has licensed or has an option to license all patents from the University of Pennsylvania that concern the use of Listeria monocytogenes or listerial products as vaccines.
